# Short term in-patient rehabilitation in axial spondyloarthritis - the results of a 2-week program performed in daily clinical practice

**DOI:** 10.1186/1756-0500-6-185

**Published:** 2013-05-07

**Authors:** Siv Grødal Eppeland, Andreas P Diamantopoulos, Dag Magnar Soldal, Glenn Haugeberg

**Affiliations:** 1 Department of Rheumatology, Hospital of Southern Norway, Servicebox 416, Kristiansand.S, 4604, Norway; 2 Department of Neuroscience, Division of Rheumatology, Norwegian University of Science and Technology, Trondheim, 7489, Norway

**Keywords:** Ankylosing spondylitis, Axial spondyloarthritis, Rehabilitation, Exercise therapy, Health services

## Abstract

**Background:**

From a health service perspective, society, with its limited resources, needs to be reassured that evidence-based medicine is also effective when carried out in the frame of ordinary clinical practice. The effectiveness of rehabilitation programs in ankylosing spondylitis (AS) has been proven to be effective in clinical trials. However, less is known when this is carried out in clinical practice. The aim of this study was to evaluate the effect of a 2-weeks rehabilitation program on self-reported outcome and physical function in patients with axial spondyloarthritis (ax-SpA) including AS patients carried out in ordinary clinical practice. The program contained of daily water exercises, exercises for flexibility, muscle strength, and cardio-respiratory fitness.

**Results:**

A total of 87 ax-SpA patients (60 men, 27 women), aged ≥ 18 years were identified to have participated in the 2-weeks in-patient rehabilitation program. Mean age was 49 years and disease duration was 14 years. 92.5% were HLA-B27 positive, 62% were current users of non-steroidal anti-inflammatory drugs, and 17% were current users of tumour necrosis factor inhibitors. After 2-weeks, a statistical significant improvement (p < 0.001) was observed for patient-reported outcomes (Bath Ankylosing Spondylitis (BAS) Disease Activity Index 4.3 vs. 3.1, BAS Functional Index 3.1 vs. 2.4) and physical measured outcomes (BAS Metrology Index 3.23 vs. 2.29, Gait Velocity 2.2 vs. 2.6 m/s, timed-stands test 22.5 vs. 16.3 s, finger-floor distance 17.9 vs. 8.9 cm, chest expansion 3.9 vs. 4.6 cm).

**Conclusion:**

Data, from our retrospective case series report, support that patient with ax-SpA benefit from short-term rehabilitation when it is carried out in ordinary clinical care. Data from ordinary clinical care may be important when discussing the effectiveness of a treatment and allocating resources in the health care system.

## Background

Axial Spondyloarthritis (ax-SpA) is diagnosed when signs and symptoms caused by chronic inflammation in the axial skeleton is present. Inflammation in the axial skeleton causes pain, stiffness and limitation of thoracic and spinal mobility. The imaging hallmark of ax-SpA is sacroiliitis visible, e.g. on radiographs or magnetic resonance imaging (MRI) and signs of new bone formations in the axial skeleton [1]. Ankylosing spondylitis (AS) is a phenotype of ax-SpA, diagnosed by the presence of sacroiliitis on radiographs, which in its final stage may cause ankylosing at sacroiliac joints and the spine [2]. Apart from pharmacological treatment (Non Steroidal Anti-Inflammatory Drugs (NSAIDs) and anti-Tumour Necrosis Factor (TNF) -α treatment), exercise and rehabilitation therapy are cornerstones in the management of ax-SpA including AS [3,4].

There is a substantial body of evidence in the literature proving that various spa and exercise programs tested in randomised trials have a beneficial effect in AS patients improving physical function and reducing symptoms of disease [5-12]. In a recent review, the authors concluded that physical therapy exercises in various modalities have positive effects on BASFI, BASDAI, pain and mobility outcomes [13]. Furthermore, in a Cochrane review, the results suggested individual home-based or supervised exercise programs to be better than non-intervention, supervised group physiotherapy to be better than home-exercise, and combined in-patient spa-exercise therapy followed by group physiotherapy to be better than group physiotherapy alone [14]. This knowledge is reflected in recommendations for management of AS, highlighting that regular exercise and rehabilitation therapy are part of the treatment strategy [3,15].

The gold standard for treatment in modern medicine is based on evidence from randomised clinical trials. However, the knowledge on the benefit and effect of a treatment and/or an intervention carried out in daily clinical care is also important and essential for patients, health care providers and decision makers in the health care system [16,17]. This can reassure that treatment based on evidence is also effective when applied in daily clinical practice.

The main aim of this study was to evaluate the short-term effect of an in-patient 2-weeks rehabilitation and exercise program on self-reported outcome and physical functions in ax-SpA patients, within the frame of standard routine and clinical care.

## Methods

In this retrospective case series report we retrieved from our hospital database ax-SpA patients who had participated in our 2-weeks in-patient rehabilitation intervention program in the period from January 2007 to June 2011. The Department of Rheumatology is the only one of its kind located in the geographic area of southern Norway with a population of approximately 200 000 inhabitants, aged 20 years and older. All included patients had to be ≥18 years of age and have a diagnosis of ax-SpA with imaging findings on X-ray, computer tomography (CT) and/or MRI confirming sacroiliitis.

All ax-SpA patients who were participating had been referred by a rheumatologist. Only patients thought to benefit from the program based on an overall clinical judgment were included in the program. Patients with severe comorbidities or severe reduced exercise tolerance were not offered participation in the program. All patients had signs and symptoms from their ax-SpA. The patients were assessed at admission and at the end of the 2-weeks program, following a standard clinical procedure for clinical assessment of ax-SpA patients at the department, which included both patient-reported outcome measures and therapist-measured outcomes. Standard routine clinical data collection and registration at the department included a broad spectre of demographics, measures of disease activity, health status and physical function, laboratory data and treatment (Table 1). Disease activity was assessed by Bath Ankylosing Disease Activity Index (BASDAI) which consists of six visual analogue scales dealing with fatigue, spinal pain, joint pain, localised tenderness, and quality and quantity of morning stiffness [18]. Functional status was evaluated by Bath Ankylosing Functional Index (BASFI) which consists of eight visual analogue scales dealing with physical function and two scales reflecting the patient’s ability to cope with daily activities [19]. Bath Ankylosing Metrology Index (BASMI) was calculated with the measurements of wall-to-tragus distance, lumbar flexion, cervical rotation, lumbar lateral flexion, and intermalleolar distance [20]. These measurements are widely used instruments in assessment of patients suffering from ax-SpA. A flexible measuring tape was used to measure mobility variables: chest expansion and finger-to-floor distance. Standardised registration in this program also included two generic instruments for physical function, the timed-stands test which measures ten repetitions sit-to-stand [21], and a 6 m maximum gait velocity test. The same physiotherapist examined the patient at admission and at discharge and performed all therapist-measured outcomes.

**Table 1:** Patient-reported outcome measures (PROMs), therapist measured outcomes and laboratory data in axial spondyloarthritis patients following a 2-weeks in-patient rehabilitation and training program

	N*	**Baseline**	**2-weeks follow-up**	P**
**PROMs**				
BASDAI, 0–10 scale	59	4.3 (2.2)	3.1 (2.1)	<0.001
BASFI, 0–10 scale	57	3.1 (1.9)	2.3 (2.0)	<0.001
**Therapist measured outcomes**				
BASMI, 0–10 scale	87	3.2 (2.4)	2.3 (2.4)	<0.001
Gait velocity, m/s	83	2.2 (0.5)	2.7 (0.6)	<0.001
Timed-stand test, s	86	21.8 (8.1)	15.8 (4.8)	<0.001
Finger-to-floor distance, cm	49	15.1 (14.0)	9.1 (12.7)	<0.001
		11.0 [25.0]	0 [16.0]	<0.001
Chest expansion, cm	46	3.7 (2.2)	4.7 (2.3)	<0.001
**Laboratory data**				
ESR, mm/hr	32	22.6 (23.4)	18.7 (15.3)	0.21
		14.0 [17.0]	16.0 [22.8]	0.44
CRP, mg/l	47	10.3 (13.7)	9.3 (10.7)	0.59
		5.0 [7.1]	4.4 [15.6]	0.71

Data are presented as mean with (standard deviation) and also with median [inter-quartile range] when data distribution were skewed.

* Values differ from 87 due to missing values.

** Paired student’s *t*-test comparing mean values and Wilcoxon test comparing median values.

BASDAI: Bath Ankylosing Spondylitis Disease Activity Index, BASFI: Bath Ankylosing Spondylitis Functional Index, BASMI: Bath Ankylosing Spondylitis Metrology Index, ESR: Erythrocyte Sedimentation Rate. CRP: C-reactive protein.

The multidisciplinary team at the rehabilitation unit consists of rheumatologist, physiotherapist, occupational therapist, social worker, and secretary. The training program, which was performed 5 days a week, was organised by an experienced physiotherapist and based on current evidence-based knowledge. The daily rehabilitation program consists of water exercises (30 minutes), basic exercises for movement, muscle strength and stability, balance and coordination (45 minutes), and exercises for endurance (40 minutes), aiming at keeping the training intensity as recommended in the literature [22]. The training is instructed by a physiotherapist in groups of four, but with an individual focus according to the individual goals and statuses of the patients. In addition, every patient has daily individual physiotherapy including massage, stretching, mobilisation/articulation and advice on specific exercises to enhance a good body posture. The patients were also followed up as needed by the other professions listed above.

We also aimed to collect data on BASDAI, BASFI and BASMI from the first follow-up at the outpatient clinic after the training program. These data were collected as part of a clinical visit at the rheumatology out-patient clinic were retrieved from the hospital database.

We used the SPSS statistical program, version 16.0 to do the statistical analysis. Normal distributed data were presented as mean with standard deviation (SD) and data with skewed distribution were also presented as median with inter-quartile range (25^th^–75^th^ percentiles).

For group comparison, we used paired Student’s *t*-test for continuous variables. If continuous variables had a skewed distribution, we also performed a non-parametric Wilcoxon test. For variables with a statistically significant difference between baseline and 2 weeks follow-up, the change from baseline to 2 weeks was also presented as a percentage change with 95% confidence intervals (CI). The physiotherapist involved in patient assessment (SGE) did not analyse the data.

The study was approved by the regional committee for ethics and medical research (REK sør-øst, Norway).

## Results

For the assessment period 95 ax-SpA patients started the 2-week rehabilitation program. Among them 8 patients (6 men and 2 women) did not complete the 2-week program for various reasons including acute infectious diseases, acute low back pain, depression and vertigo. A total of 87 ax-SpA patients were thus eligible for evaluation (60 men and 27 women). Mean (SD) age was 49.2 (10.0) years, body mass index (BMI) was 26.7 kg/m2 (3.5), education was 12.2 (4.0) years, and disease duration was 14.4 years (11.9). Among the patients, 92.5% were HLA-B27 positive and 27.2% were smoking. Furthermore, 50.6% were employed, 9.2% had combined disability pension and were employed part-time and 29.9% had a disability pension. Among the patients, 62.1% were current users of NSAIDs and 17.2% of TNF inhibitors (10 etanercept, 3 infliximab and 2 adalimumab). All patients fulfilled the Assessment of SpondyloArthritis international Society (ASAS) criteria for axial spondyloarthritis [1]. All patients had imaging evidence of sacroiliitis. Among the 72 patients with available radiographs of the sacroiliac joint 64 patients had radiographic sacroiliitis and fulfilled the modified New York criteria for AS [2]. Among the 23 patients not fulfilling the New York criteria for AS, 18 patients had sacroiliitis on MRI and 5 patients had sacroiliitis on CT.

As shown in Table 1, a statistical significant improvement (p < 0.001) was observed both for patient-reported and physical-measured outcomes after 2 weeks. Expressed as percentages (95% CI), the improvement for mean BASDAI was 27% (18–37%), for BASFI 26% (15–37%), for BASMI 29% (22–36%), for gait velocity 20% (17–23%), for timed-stand test 27% (23–32%), for finger-to-floor distance 40% (26–53%) and chest expansion 26% (19–33%). However, no significant differences were seen for ESR (16.5 vs. 16.9 mm/hr) and CRP (8.2 vs. 8.3 mg/l).

Among the 48 ax-SpA patients with retrievable follow-up data, no significant differences were seen in a mean time-period of 9.3 months (SD 6.9) after the training program for BASDAI [mean 4.4 (2.2) vs 4.1 (2.3), p = 0.24] and BASFI [mean 3.5 (2.6) vs 3.2 (2.0), p = 0.31]. However, BASMI at follow-up remained significantly improved, compared with the baseline [mean 2.7 (2.5) vs 3.3 (2.6), p = 0.02].

## Discussion

Our study supports that patients with ax-SpA, benefit from structured and intensive exercise training programs, and also when these are carried out in the setting of ordinary clinical practice. In our study, ax-SpA patients improved self-reported disease activity by 27% (BASDAI) and function by 26% (BASFI), whereas improvement in physical measured function ranged from 20% for gait velocity to 40% for finger-to-floor distance.

Various exercise and rehabilitation programs proving efficacy have been tested in randomised trials [5-12]. This includes different exercise programs [9,10,12], spa rehabilitation and exercise programs [6,7], combined intensive group exercise with an educational-behavioural program [8], and rehabilitation and exercise programs tested in different climates [11]. In a Cochrane review of the literature, it was concluded that supervised group therapy was better than home exercises, and combined in-patient spa-exercise therapy followed by group physiotherapy was better than group physiotherapy alone [14]. In our study, exercises were carried out in groups.

In the study by Strumse et al., comparing a 4-weeks rehabilitation program in Mediterranean and Norwegian settings [11], patients in the Norwegian environment reduced their BASDAI by 1.9 from a baseline of 5.8 (33%) and BASFI by 1.2 from a baseline of 4.3 (28%) which is in the same percentage range as in our study. Patients, however, in the Mediterranean setting had a significantly higher reduction than for patients in the Norwegian setting with a reduction in BASDAI by 4.2 from a baseline of 6.2 (68%) and BASFI by 2.6 from a baseline of 4.3 (60%). The results of this study indicate that warm climate does have an additional effect on rehabilitation and exercise programs for the AS patients. The baseline values in this study were, however, higher than in our study.

Pharmacological treatment with NSAIDs [23] and TNF inhibitors [24-26] has proven to improve signs and symptoms and to improve physical function in AS. NSAIDs are considered as first-line in pharmacological treatment of AS, whereas, TNF inhibitors are recommended used in patients with active disease when exercise, and physiotherapy and NSAIDs have failed [3]. In our study, 62% were NSAID and 17% were current users of TNF inhibitors. In the ASSERT-infliximab study, evaluating the efficacy of infliximab in AS patients with active disease at inclusion (BASDAI ≥ 4), patients improved in median their BASDAI by 2.9, BASFI by 1.7 and BASMI by 1.0 scale points during 24 weeks of follow up, reflecting a percentage improvement by, approximately, 45% for BASDAI, 30% for BASFI and 21% for BASMI [24]. The same level of improvement rates has also been reported for other TNF inhibitors, e.g. adalimumab and etanercept [25,26]. Interestingly, the corresponding figures for percentage changes for BASDAI, BASFI and BASMI in our study were in the same range as for these studies exploring the effect of TNF inhibition. This emphasises and illustrates the effectiveness of exercise programs in AS patients.

In the randomised, controlled study by Masiero et al., was demonstrated that even AS patients stabilised on TNF inhibitors were able to further improve spine mobility and reduce pain, stiffness, and disability by a rehabilitation program based on an educational-behavioral intervention and exercise training program [8]. In the study by Colina et al., an intensive rehabilitation program in AS patients treated with the TNF-inhibitor etanercept was shown to contribute to reducing disability and to ameliorating quality of life [6]. These points highlight that exercise programs should be part of the treatment strategy, even in patients who have responded favourably to anti-TNF treatment. The importance of exercise programs in AS is further highlighted by evidence suggesting that AS patients are at increased risk of cardiovascular diseases [27]. In a recently published cross-sectional study, AS patients were found not only to have reduced flexibility, but also to have lower cardio-respiratory fitness, again underlining the importance of exercising in this patient group [28].

Exercise programs have also been proven to be cost effective. A randomised controlled trial showed that combined spa-exercise therapy with drugs and weekly group physical therapy had a more favourable cost-effectiveness and cost-utility compared with patients who stayed at home and continued their usual activities and standard treatment [29].

One of the big challenges is patient’s adherence to the exercise program [30]. In a recent publication, evaluating exercise programs, it was found that only four trials reported on participants’ adherence to the exercise programs and that only one provided sufficient information to evaluate the possible influence of the adherence [30]. Our data indicate that the positive effects on self-reported disease activity (BASDAI) and function (BASFI) gained from the training program are lost and diminished after the training program is stopped. In a recently published prospective Norwegian study, it was found that patients were back to their base-line health status six months after discharge from rehabilitation [12]. This highlights the importance of supporting patients and encouraging them to continue exercising and also, after finishing intensive exercise programs, to maintain the levels of benefits that have been achieved.

One of the major challenges comparing the results between rehabilitation studies is the lack of standardisation and the variation in the content, exercise intensity and duration. In our study, the intervention period was 5 days a week for 2 weeks, whereas, e.g. in the study by Colina et al., [6] the intervention period was one week and in the study by Ince et al., the period was three months [10]. In our study, the daily program consisted of water exercises (30 minutes), basic exercises for movement, muscle strength and stability, balance and coordination (45 minutes), and exercises for endurance (40 minutes). In addition, every patient had daily individual physiotherapy including massage, stretching, mobilisation and articulation, and advice on specific exercises to enhance a good body posture. In the study by Ince et al., the exercise program consisted of 50 minutes of multimodal exercise, including aerobic, stretching, and pulmonary exercises, 3 times a week for 3 months [10]. Whereas, in the study by Masiero et al., the program consisted of 12 rehabilitation exercise sessions (stretching, strengthening, chest and spine/hip joint flexibility exercises), which patients performed at home [8].

The limitations of our study include both selection bias of participants and study design. The study design was retrospective, based on data collection as part of clinical routine. Despite the clinical nature of this program and registration of variables which were standardised, data were missing in a high proportion of patients, particularly for some variables (e.g. BASDAI and BASFI). This emphasises that there is a need for more attention and focus, in clinical practice, to use recommended outcome measures in the follow-up and assessment of patients with inflammatory rheumatic disorders. In the updated 2010 ASAS/EULAR recommendations for the management of AS, disease monitoring of patients with AS is recommended to include data on e.g. patient history (e.g. questionnaires), clinical parameters and laboratory tests [15]. Our experience demonstrates that disease monitoring in clinical practice is facilitated and has improved in our hospital after we started to use the GoTreatIT-rheuma® computer system (http://www.diagraphit.com), designed to monitor patients, in daily clinical practice, with inflammatory joint disorders [31]. Furthermore, the ax-SpA patients we assessed were not a random selection of the ax-SpA population, followed in our out-patient clinic. Patients who did not have e.g. sufficient effect of medical treatment were more likely recruited. Another limitation, was the inclusion criteria which was only based on an overall judgement by the referral rheumatologist, that the patients were thought to benefit from this program. The lack of a control group also limits the scientific validity of the data, as the placebo effect was not controlled for and further includes both patient-reported outcome measures and therapist-measured outcomes. Another limitation of this study is that no intra-rater reliability tests were performed for the measurements used. However, for the metric measurement, BASMI, and for the patient reported outcome measures, BASDAI and BASFI, the instruments have been shown to be reliable, valid and sensitive to change [20,32].

The strength of our study is that data have been captured from real life and based on how the ax-SpA patients have been handled in ordinary clinical practice. Thus, the design of this study enables us to test evidence-based treatment recommendations [14] and to validate if the effect of rehabilitation can also be demonstrated in clinical practice, after demonstration in clinical trials. Our data add evidence that organised and structured physiotherapy is of benefit for the ax-SpA patients. Probably, it has also been of advantage that all measures of physical function were performed by experienced physiotherapists, thus, reducing the risk of measurement errors.

Interestingly, our data indicate that the positive effect on BASMI, which is a composite physical measure of physical function, is also maintained after weeks and months after the training program is stopped. This positive findings should be interpreted with caution, as the follow-up measures of function for BASMI were not performed by the same assessor as during the 2-weeks training program.

## Conclusion

Data from our retrospective case series report, support that patients with ax-SpA, benefit from short-term rehabilitation when carried out in ordinary clinical care. From a health service perspective “reality-based studies” are important when discussing the effectiveness of a treatment and allocating resources in the health care system.

## Competing interests

The authors declare that they have no competing interests.

## Authors’ contributions

All authors contributed to the conception and design of the study, interpretation of the data, and revision of the final manuscript. GH was the principal investigator. All authors read and approved the final manuscript.
